# Evaluating Dysfunction in Fever-Induced Paroxysmal Weakness and Encephalopathy

**DOI:** 10.3390/children10040703

**Published:** 2023-04-10

**Authors:** Fumikazu Sano, Toshimichi Fukao, Hideaki Yagasaki, Hideaki Kanemura, Takeshi Inukai, Yoshimi Kaga, Takaya Nakane

**Affiliations:** Department of Pediatrics, Faculty of Medicine, University of Yamanashi, 1110 Shimokato, Chuo, Yamanashi 409-3898, Japan

**Keywords:** *ATP1A3*, heterozygous variants, FIPWE, RECA, electroencephalogram, nerve conduction studies

## Abstract

Heterozygous variants in the *ATP1A3* gene are linked to well-known neurological phenotypes. There has been growing evidence for a separate phenotype associated with variants in residue Arg756—fever-induced paroxysmal weakness and encephalopathy (FIPWE) or relapsing encephalopathy with cerebellar ataxia (RECA). With only about 20 cases being reported, the clinical features associated with mutations at Arg756 have not been fully elucidated. We report a case of FIPWE with a p.Arg756Cys change in the *ATP1A3* gene and a comparison of the clinical features, including electrophysiological examination, with previous cases. The 3-year-old male patient had normal psychomotor development, presenting with recurrent symptoms of generalized hypotonia with loss of gait, mutism, and dystonic movements only during febrile illnesses since 19 months of age. At 2.7 years of age, a third neurological decompensation episode occurred, during which electroencephalography (EEG) did not reveal high voltage slow waves or epileptiform discharge. Nerve conduction studies (NCS) also did not show latency delay or amplitude reduction. *ATP1A3* exon sequencing showed a heterozygous p.Arg756Cys mutation. While the patient experienced repeated encephalopathy-like episodes, including severe hypotonia during febrile illness, EEG and NCS did not reveal any obvious abnormalities. These electrophysiological findings may represent an opportunity to suspect FIPWE and RECA.

## 1. Introduction

The *ATP1A3* gene codes for the α-3 catalytic subunit of the Na^+^/K^+^-ATPase pump, which regulates neuronal resting potential. Heterozygous mutations in the *ATP1A3* gene cause several well-characterized neurologic syndromes: alternating hemiplegia of childhood, rapid-onset dystonia parkinsonism (RDP), and CAPOS (*c*erebellar ataxia, *a*reflexia, *p*es cavus, *o*ptic atrophy, and *s*ensorineural hearing loss) syndrome [1]. To date, *ATP1A3* mutation-related disorders have been shown to have distinct phenotypes suggesting a unique pathophysiological response to the related *ATP1A3* mutations. Similarly, novel phenotypes have been described for the heterozygous missense mutations at Arg756, which usually present as fever-induced paroxysmal weakness and encephalopathy (FIPWE) [2] or relapsing encephalopathy with cerebellar ataxia (RECA) [3]. As only approximately 20 cases have been reported [2,3,4,5,6,7,8,9], the clinical features associated with mutations at Arg756 have not been fully elucidated. To clarify the pathophysiology of weakness and encephalopathy episodes, we report a case of FIPWE caused by Arg756Cys mutation in *ATP1A3* and comparison of the clinical features with previous cases, particularly the electrophysiological outcomes.

## 2. Case Presentation

**Figure 1:**
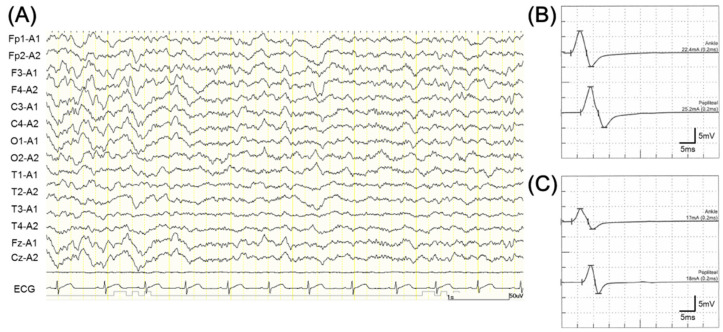
Electrophysiological data from the patient on admission at the age of 2.7 years. (**A**) Electroencephalogram (EEG) on day 2 after admission. Although the patient presented a less responsive status to external stimuli, EEG did not reveal comatose encephalopathy. (**B**,**C**) Nerve conduction study of tibial nerve ((**B**) left; (**C**) right) did not show latency delay or amplitude reduction.

**Figure 2:**
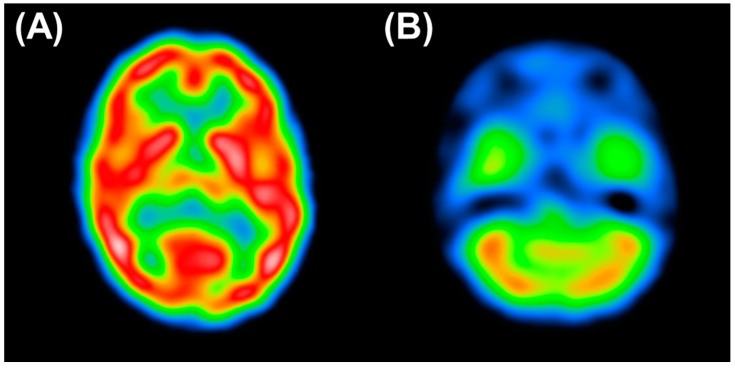
Cerebral blood flow (CBF) evaluated by ^99m^Tc-ethyl cysteinate dimer single photon emission computed tomography (SPECT). (**A**) No significant CBF abnormality in the cerebrum and basal ganglia is seen. (**B**) SPECT shows mild decrease in CBF in the cerebellum.

The patient was a 3-year-old boy who was the second among two children from healthy non-consanguineous Japanese parents. He was born following a normal pregnancy without asphyxia or other perinatal events. He had regular psychomotor development until 19 months of age. The patient suffered from a viral infection with fever at 19 and 21 months of age. During those periods of febrile illness, he presented with generalized hypotonia with loss of gait and mutism without altered consciousness. He recovered slowly, and within 9 months was able to sit unassisted. At 2.7 years of age, during a febrile episode of adenovirus enteritis, a third neurological episode occurred, with severe hypotonia, ataxia, dyskinesia of the trunk and upper limbs, swallowing disorder, mutism, and less responsive dystonic movements of the bilateral upper and lower limbs with irritable state. Chorea athetosis was not observed. Routine electroencephalogram (EEG), recorded during awake and natural sleep (stage 1), did not show any high voltage slow wave epileptiform discharge. A photic stimulation showed no significant findings, and hyperventilation was not performed because the patient was unable to follow instructions. Nerve conduction studies (NCS) and auditory evoked potential did not show latency delay or amplitude reduction (Figure 1). Laboratory diagnostic workup, including metabolic investigations and cerebrospinal fluid (CSF) examination, revealed a slight increase in serum and CSF lactate (47.8 and 23.7 mg/dL, respectively) and pyruvate (2.84 and 1.40 mg/dL, respectively) levels. Plasma amino acid analyses revealed elevated plasma alanine levels (497.8 nmol/mL) and increased alanine-to-lysine ratio (Ala/Lys molar ratio = 4.1). Brain and spine magnetic resonance imaging (MRI) and skeletal muscle computed tomography (CT) showed no abnormalities. ^99m^Tc-ethyl cysteinate dimer single photon emission computed tomography (SPECT) showed no significant abnormalities in cerebral blood flow in the cerebrum (right: 57.9 mL/100 g/min and left: 53.4 mL/100 g/min) and basal ganglia (right: 60.1 mL/100 g/min and left: 59.5 mL/100 g/min). However, mild hypoperfusion was observed in the cerebellum (right: 49.5 mL/100 g/min and left: 50.2 mL/100 g/min) (Figure 2). L-carnitine and thiamine were administered, and the patient did not experience any recurrent episodes during the febrile illness. At his most recent follow-up (at 3.5 years of age), he had gradually recovered partially but still had impaired language, generalized mild hypotonia, ataxia, dyskinesias of the trunk and upper limbs, and dystonia of the bilateral upper and lower limbs.

*ATP1A3* exon sequencing showed a heterozygous mutation (Arg756Cys). The variant was classified as pathogenic because the same amino acid alternation has previously been identified in cases with similar phenotypic features.

## 3. Discussion

The α3 subunit of the Na^+^/K^+^-ATPase pump, which is the product of the *ATP1A3* gene, participates in neuronal resting potential by maintaining the sodium/potassium gradient across the cell membrane. α3 subunit is selectively expressed in neurons and highly enriched in the basal ganglia, thalamus, and cerebellum [10]. Although the mechanisms of pathogenesis are unknown, the anatomical distribution of α3 may correlate with neurological deficits seen in clinical phenotypes, such as *ATP1A3*-related disorders, including FIPWE and RECA.

Altered consciousness, lack of reaction, bradykinesia, and mutism are sometimes reported in cases of FIPWE and RECA [2,9,10,11,12,13,14,15,16,17]. However, little is known about the pathophysiology of encephalopathy-like episodes, particularly altered consciousness with FIPWE and RECA. The patient, in this case, was also irritable and less responsive to neurological examinations or environmental stimuli. However, EEG recordings showed regular background activity with no encephalopathy-like slow waves in the acute phase of the episode. These EEG findings strongly suggest that cortical dysfunction was unlikely the underlying cause for the lowered responsiveness. Since EEG abnormalities are often lacking in reported cases (Table 1), the transiently altered state of consciousness represented by unresponsiveness and mutism is likely due to thalamic or cerebellar dysfunction rather than cortical dysfunction, considering the distribution of ATP1α3.

There are few reports on cerebral blood flow in *ATP1A3*-related diseases; a ^99m^Tc- hexamethylpropylene amine oxime scan showed normal CBF in RDP [18]. Although this case was considered to be of FIPWE, there was a slight decrease in cerebellar blood flow, but no significant abnormal findings were observed in the cerebrum, as in previous reports of RDP. The SPECT findings also suggest that the pathophysiology of the lowered responsiveness seen in FIPWE may involve cerebellar dysfunction rather than cortical dysfunction.

Severe hypotonia and flaccid paralysis during febrile illness seems to be a common symptom in FIPWE and RECA caused by mutations at the Arg756 position [2,9,10,11,12,13,14,15,16,17]. However, the pathophysiology of hypotonia with FIPWE and RECA is not well described. In our case, the patient showed severe hypotonia and flaccid paralysis, while NCS, CSF, serum creatine kinase, and skeletal muscle CT did not present abnormalities, in line with previous reports (Table 1). From these findings, muscles and peripheral nerves were unlikely to be involved in the hypotonia. Although significant findings, such as cerebral blood flow abnormalities, cannot be ruled out, basal ganglia, including the internal capsule, may contribute to hypotonia and flaccid paralysis episodes in FIPWE and RECA rather than peripheral nerves.

As reported earlier and in our case, in FIPWE and RECA, it may be difficult to observe characteristic findings by conventional MRI or electrophysiological examination in only the acute phase. In order to understand the pathophysiology of FIPWE and RECA in more detail, it may be necessary to perform these assessments successively through the clinical course.

## 4. Conclusions

Though the patient in the present study with an *ATP1A3*-associated Arg756Cys mutation in experienced repeated encephalopathy-like episodes, including severe hypotonia during febrile illness, EEG and NCS did not present any obvious abnormalities. These electrophysiological features may be an opportunity to suspect FIPWE and RECA. Further studies are needed to characterize the features of electrophysiological examination in *ATP1A3*-related disorders caused by mutations at the Arg756 position, hopefully leading to better diagnostic strategies for this extremely rare disease.

## Figures and Tables

**Table 1:** Clinical characteristics of patients with mutation in *ATP1A3* gene at residue 756.

Reference	Present Case	[4]	[5]	[3]	[13]	[6]	[7]	[2]	[12]
Publication year		2012	2014	2015	2015	2016	2016	2017	2017
Number of patients	1	1	1	1	1	1	1	5	2
Mutation	R756C	R756H	R756H	R756C	R756H	R756C	R756C	R756H 3/5 R756L 2/5	R756C
Age at onset	1y7m	9m	9m	1y1m	10y	1y5m	11m	13m–3y	9m–1y10m
Symptoms									
Unresponsive	+	Anarthria	Anarthria	+	-	+	+	5/5	1/2
Hypotonia	+	+	+	+	-	+	+	5/5	2/2
Mutism	+	+	-	-	-	-	-	0/5	0/2
Dystonia	+	+	+	+	+	+	+	3/5	2/2
Ataxia	-	+	-	+	+	-	+	5/5	2/2
Electrophysiological examination									
EEG	Normal	NA	Normal	Normal	Normal	Normal	Normal	Normal 1/4 Slow wave 3/4	NA
NCS	Normal	Normal	Normal	NA	NA	NA	NA	Normal 3/4 Absent F wave 1/4	NA
ABR	Normal	NA	Normal	Normal	NA	NA	NA	NA	NA
VEP	NA	NA	NA	Normal	NA	NA	NA	NA	NA
SEP	NA	NA	Normal	Normal	NA	NA	NA	NA	NA
**Reference**	**[14]**	**[15]**	**[16]**	**[8]**	**[9]**		**[11]**		**[17]**
Publication year	2017	2017	2018	2018	2019		2021		2021
Number of patients	1	3	2	1	3		2		2
Mutation	R756H	R756H	R756C	R756C	R756H 2/3 R756C 1/3		R756H		R756C
Age at onset	1y2m	8m–5y	1y6m–1y7m	9m	1y5m–5y6m		1y3m–1y7m		
Symptoms									
Unresponsive	+	0/3	1/2	+	1/3		2/2		0/2
Hypotonia	+	3/3	2/2	+	2/3		2/2		2/2
Mutism	-	0/3	0/2	-	3/3		1/2		0/2
Dystonia	+	2/3	1/2	+	1/3		0/2		1/2
Ataxia	+	3/3	2/2	+	2/3		2/2		2/2
Electrophysiological examination									
EEG	generalized epileptic discharges	NA	NA	Normal	Normal 3/3		NA		Normal 1/2
NCS	NA	NA	Normal 1/2	NA	NA		NA		NA
ABR	NA	NA	NA	NA	NA		NA		NA
VEP	NA	NA	NA	NA	NA		NA		NA
SEP	NA	NA	Normal 1/2	NA	NA		NA		NA

EEG: electroencephalography, NCS: nerve conduction study, ABR: auditory brainstem response, VEP: visual evoked potential, SEP: Somatosensory evoked potential, +: Showed symptoms, -: Did not show symptoms.

## Data Availability

Not applicable.
